# Perceived barriers and facilitators to family and community nurses’ care for older adults: a descriptive qualitative study

**DOI:** 10.1186/s12912-025-03890-4

**Published:** 2025-10-10

**Authors:** Isabella Santomauro, Erika Bassi, Erica Busca, Ines Basso, Elisa Ambrosi, Melania Stedile, Angela Durante, Alberto Dal Molin

**Affiliations:** 1 Department of Translational Medicine, University of Piemonte Orientale, Via Solaroli 17, Novara, 28100 Italy; 2 Department of Diagnostics and Public Health, University of Verona, Strada le Grazie 8, Verona, 37134 Italy; 3 Tuscany Foundation “G. Monasterio”, Via Giuseppe Moruzzi 1, Pisa, 56124 Italy; 4 Health Science Interdisciplinary Center, Scuola Superiore Sant’Anna, Piazza Martiri della Libertà 33, Pisa, 56127 Italy

**Keywords:** Family and community nursing, Older adults, Primary care, Descriptive qualitative study

## Abstract

**Background:**

The aging population is becoming a growing challenge for health care systems, pushing toward the need for systemic adaptations. In Italy, the role of the Family and Community Nurse (FCN) was introduced as a response to these demographic changes, aimed at ensuring personalized and integrated care for the older adults at the national level. Despite national institutional recognition, the implementation of the FCN role varies considerably across regions, resulting in uneven and fragmented service delivery. To better understand this variability, the present study aimed to describe the perceived barriers and facilitators influencing the delivery of community-based care to older adults.

**Methods:**

A qualitative descriptive study was conducted, from December 2023 to May 2024, using semi-structured interviews on a purposive sample. Forty-one FCNs from four Italian regions were interviewed. The interviews were transcribed verbatim and analyzed using Qualitative Content Analysis, supported by NVivo software.

**Results:**

Under the overarching theme reflecting the barriers and facilitators that influence the delivery of family and community nursing care to older adults, six sub-themes and thirteen categories were identified. The key barriers were the misalignment between strategic leadership level and managerial and operational levels, shortages of instrumental and human resources, heterogeneity and fragmentation of nursing documentation, lack of uniformity of the training pathways and, limited recognition of the FCN role. The most frequent facilitators were current regulations and national policies, the support and collaboration of local organizations and local political institutions, as well as the expansion of interprofessional collaborations.

**Conclusions:**

The study emphasizes the need for targeted interventions to strengthen the governance of the FCN role, ensuring greater integration between policy directives, resources, training, and organizational practice. This requires coordinated efforts among health care institutions, universities, and professional organizations to create a system that not only adequately trains FCNs but also ensures their functional and effective placement within community-based care.

**Clinical trial number:**

Not applicable.

## Background

More than 40% of older adults in Europe live with at least two chronic conditions, and over half of the additional life years after age 65 are spent with disability or reduced functional capacity [1]. These data underscore the increasing complexity of care needs among aging populations and the growing demand for accessible, coordinated, and person-centered healthcare services. Population aging is a well-documented global trend with far-reaching implications for health and social care systems. According to the World Health Organization (WHO), the number of people aged over 60 is expected to reach 2.1 billion by 2050 [2]. In Europe, individuals aged 65 years and older are expected to account for nearly 30% of the population by 2050, up from over 21% in 2023 [1]. In Italy, the demographic shift is particularly pronounced: as of 2023, over 24% of the population is aged 65 or older, and this proportion is expected to exceed 30% within the next two decades [3]. In light of these trends, there is a pressing need to reorient healthcare systems away from reactive, disease-focused models toward integrated and proactive approaches that promote healthy aging, especially in community-based settings. Among these, integrated care interventions have emerged as one of the most promising strategies to enhance access, continuity, and coordination within primary care services [4]. To support this transformation, health policies must recognize and strengthen the contribution of healthcare professionals with advanced competencies operating within community-based settings [1]. In this context, the Family and Community Nurse (FCN) has emerged as a key role. Since its conceptual definition by the WHO, the FCN has been described as a professional role oriented towards prevention, health promotion, and continuity of care across the life course, particularly for older adults [5]. In Italy, the FCN has been formally recognized as a strategic component in the reorganization of territorial healthcare through Ministerial Decree 77/2022 [6], with further specifications provided in the national guidelines published by National Agency for Regional Health Services (AGENAS) [7]. The FCN operates within a preventive and community-based framework, focusing on proximity, early identification of frailty, promotion of self-care and continuity of care, particularly for older and chronically ill patients. Unlike nurse practitioners in other countries such as Canada, the United States, or the United Kingdom who are typically authorized to perform diagnostic, prescriptive, and autonomous clinical tasks within primary care, the FCN does not carry out responsibilities reserved for medical professionals [8]. Rather, this role contributes to integrated, person-centered care by strengthening local service networks and responding to the complex needs of populations.

Despite national institutional recognition, the implementation of the FCN model in Italy remains uneven and fragmented. Regional initiatives have led to diverse pilot programs and organizational models [9], with variable levels of integration into primary care systems [10, 11]. Moreover, only a limited number of methodologically rigorous studies have investigated the lived experiences of FCNs across different regional contexts [11, 12]. Available evidence suggests that full implementation of new nursing role in primary care settings is influenced not only by regulatory framework, but also by team-level and organizational factors such as interprofessional collaboration, institutional recognition, responsiveness to local needs, and the capacity to effectively support older adults with complex care requirements [13, 14].

Understanding these factors from the perspective of FCNs directly engaged in community-based services is essential to inform the strategic development of the role, strengthen its operational effectiveness, and support the delivery of person-centered, coordinated care. Accordingly, this study aims to describe, through a qualitative approach, the perceived barriers and facilitators influencing the delivery of community-based care to older adults as reported by FCNs in the Italian context.

## Methods

### Design

A qualitative descriptive design was used, which allows for clear and direct descriptions of participants’ experiences and perceptions [15]. This is particularly appropriate for investigating underexplored phenomena, as it acknowledges the subjective nature of experiences and presents findings using language that closely reflects participants’ perspectives [16, 17, 18].

This approach emphasizes the description of the “what” and the “where” of experiences [19] while examining professionals’ perspectives on the barriers and facilitators influencing the delivery of community-based care to older adults within its organizational context. Semi-structured interviews were conducted to explore and understand these factors, and to facilitate their comprehensive description.

The study was conducted and reported in accordance with the *Standards for Reporting Qualitative Research* (SRQR) [20].

### Sampling and recruitment

Purposive and snowball sampling [21] were used to recruit participants between December 2023 and May 2024. Given the variability in the implementation of Family and Community Nursing across the country, maximum variation sampling at the regional level was also applied to ensure representation from Northern, Central, and Southern Italy. This geographic categorization, commonly used in national demographic and health data, captures long-standing economic and socio-cultural differences [22]. The macro-regions of Northern, Central, and Southern Italy vary in terms of population ageing dynamics, access to health and social care services, and overall socio-economic status, all of which may shape the barriers and facilitators experienced by FCNs within each area. The research team contacted the Association of Family and Community Nurses (AIFeC: https://www.aifec.it/), Italy’s national professional association for FCNs, to disseminate the study invitation among its members. The association brings together FCNs working in clinical practice as well as in managerial roles within community health services. Participants were contacted by phone and/or e-mail and received a detailed explanation of the study. They were asked to sign the informed consent form and subsequently provided their availability to schedule a Voice-over Internet Protocol (VoIP) interview at a convenient time and date individually.

### Inclusion criteria

The inclusion criteria for participants were being employed as an FCN within community health services. In addition, participants were asked to confirm that they had the necessary technological resources to participate in an online interview.

### Data collection and instruments

Semi-structured interviews were conducted by a female nurse researcher with extensive experience in qualitative research (AD), supported by a trained researcher from an external research organization with long experience in sociological studies. Additionally, a nurse doctoral student who was trained in qualitative research (IS) contributed to the process by scheduling interviews. All interviews were conducted in the native local language, and audio-recorded via Google Meet, a video communication service developed by Google.

The interview guide developed following the structure proposed by Roberts [23] was piloted with three individuals who were similar to the target population prior to its use [24]. Before starting the interview, participants were provided with a clear explanation of its purpose. The first section of the interview included questions aimed at exploring their general workday as FCN. The second section focused specifically on describing barriers and facilitators influencing the adoption and the delivery of family and community nursing care within the organizational context. The interview guide is detailed in Box 1. Following the interview, participants completed a questionnaire to collect socio demographic information. Digitally recorded interviews were transcribed verbatim using Nvivo transcription software (version 12). To assess accuracy, the nurse doctoral student spot-checked 24% of the transcripts [25].

**Table:** 

^Box 1. Interview guide^	
a) Can you describe a routine workday in your role? b) What difficulties or barriers influence the delivery of nursing care by the FCN? c) In your opinion, what are the facilitating factors in performing the role of FCN?	

### Data analysis

Data were analyzed using Qualitative Content Analysis that is a systematic coding and categorizing approach used for exploring large amounts of textual information unobtrusively to determine trends and patterns of words used and the discourse of communication [26]. Two researchers (IS and MS) independently read and re-read the interview transcripts to become familiar with the data. Through an inductive process, meaningful units of text were identified and coded to reflect key elements of participants’ narratives. NVivo^®^ software (version 12) was used to support systematic coding. A subset of transcripts (*N* = 10) was independently coded by both researchers to ensure consistency; any discrepancies were resolved through discussion with a third member of the research team that conducted the interviews (AD). The resulting codes were refined to minimize overlap and then organized into broader, mutually exclusive categories, which were subsequently grouped into sub-themes based on recurrence, structural similarities, and logical connections. Sub-themes were further reviewed and refined collaboratively within the research team. Code saturation was monitored throughout the data analysis until no new significant codes emerged and the codebook demonstrated stability [27]. This process ensured that the identified categories were sufficiently representative to address the study’s objective. To preserve the cultural and linguistic nuances of participants’ accounts, all interviews were transcribed and analyzed in the original language (Italian). Two team members (IS and EB) performed translation and back-translation to ensure rigor [28]. Both translators have a comprehensive understanding of both the source and target languages, as well as the two cultures. Finally, two authors (IS and EB) compiled the emerging sub-themes into a report to present the findings, incorporating quotes (interview number, sex, and age) as illustrative evidence for each sub-theme.

### Ethical considerations

The study respected the principle of the Helsinki Declaration. Ethical approval was obtained from the Novara Ethics Committee (n° CE307/2023). Participants were assigned unique identification codes to anonymize their responses, and all personal identifiers were removed from transcripts. The recordings were securely stored on the University of Piemonte Orientale server.

### Rigor

Rigor was ensured through a range of strategies consistent with established criteria for trustworthiness in qualitative research [29]. Credibility was strengthened by the involvement of a multidisciplinary research team with expertise in qualitative methods and nursing practice, who worked collaboratively throughout the analytic process. Independent coding, peer debriefing, and member checking contributed to enhancing the credibility and accuracy of the findings [30]. Specifically, member checking was conducted at the end of each interview to confirm the accuracy and resonance of participants’ accounts with their lived experiences [30].

Dependability and confirmability were supported through regular team meetings to reflect on emerging findings and document analytical decisions.

Transferability was promoted through purposive and maximum variation sampling, as well as the provision of contextual details to support the assessment of applicability in similar settings by external readers.

## Results

### Study participants

Forty-one participants meeting the inclusion criteria were interviewed. They represented four Italian regions: two in Northern Italy (North-West and North-East), one in Central Italy, and one in Southern Italy and reflected various experiences in Family and Community Nursing practice. The majority of participants were female (*n* = 36, 88%), with a mean age of 43 years (SD ± 8.4; [26–59]). All 41 participants were employed in FCN roles, including four who also held managerial positions. All participants worked in public community health service facilities as Local Health Authorities and Community Health Centers. These health service facilities provide a range of primary and community-based services, including general medical care, health promotion and prevention programs, nursing care, home visits, management of chronic conditions, and support for vulnerable or elderly patients.

The participants had heterogeneous educational backgrounds due to the variability of training level at the regional level; the majority (*n* = 37, 90%) held a postgraduate degree in Family and Community Nursing (60 ECTS – European Credit Transfer and Accumulation System).

The interviews lasted an average of 50 min (range: 35–70 min), excluding time for introductions and greetings.

The main sociodemographic and professional characteristics are reported in Table 1.

**Table 1:** Characteristics of participants

Variable	Participants (*N* = 41)
Female (*n*, %)	36 (88)
Age, mean (± SD) [min-max]	43 ± (8.4) [26–59]
Regions (*n*, %)	
North-West	24 (58.4)
North-East	6 (14.7)
Central	6 (14.7)
South	5 (12.2)
Overall work experience in year, mean (± SD) [min-max]	19.6 ± 9.6 [4–37]
Training level in Family and Community Nursing	
Postgraduate degree	37 (90.2)
Specialized clinical course	4 (9.8)
Choice to become FCNs	
Yes	29 (70.7)
No	12 (29.3)
Intention to continue as FCNs*	
Yes	37 (90.2)
No	3 (7.4)
*Missing	

### Barriers and facilitators

Under the overarching theme *“*Factors influencing the delivery of Family and Community Nursing care” six sub-themes and thirteen categories were identified, reflecting participants’ experiences and perceptions of the key barriers and facilitators influencing the delivery of family and community nursing care in the regions involved (Table 2).

The sub-themes were organized according to a conceptual structure, with the aim of supporting a clearer presentation and potentially enhancing the overall readability of the findings. The conceptual structure employed was drawn from the scoping review by Nilsen and Bernhardsson [31], in which the dimensions characterizing implementation processes are organized into four distinct levels, including micro, meso, and macro levels, as well as a multiple level capturing cross-cutting influences of healthcare systems. The macro level refers to broad external influences such as political, regulatory, and sociocultural factors; the meso level concerns organizational and managerial elements operating at the local level; the micro level includes patient-related influences; and the multiple level encompasses relational, cultural, and resource-related aspects that span across levels and cannot be attributed to a single domain.

In this study the macro level comprised the wider environment sub-theme. Facilitating factors included supportive national policies, regulatory frameworks, local political institutions, and the presence of community-based organizations, all of which shaped the broader system in which FCNs operate.

At the meso level, two sub-themes were identified: organizational readiness to change and organizational support. Leadership engagement and the availability of specific training opportunities for FCNs were considered as facilitators, while barriers included misalignment between strategic objectives and operational practices, the limited formal recognition of the FCN role within organizations, and the persistent shortages of staff, transportation, and equipment.

At the multiple level, several cross-cutting influences emerged. Financial constraints represented a challenge with the lack of dedicated financial incentives undermining the sustainability and institutionalization of the FCN role. The sub-theme of feedback systems highlighted fragmented documentation and inadequate evaluation processes, perceived as obstacles to service improvement and accountability. Role recognition was also described as a complex area: although informal acknowledgment and interprofessional collaboration occasionally supported role visibility, these were often counterbalanced by a general lack of awareness, variability in training pathways, and the continued predominance of a medically-centered model of care.

No sub-themes or categories that emerged in this study could be mapped to the micro-level dimension related to patient-level determinants. Participants’ accounts were primarily focused on organizational, structural, and cross-cutting contextual factors influencing the delivery of family and community nursing.

This conceptual structure served as a useful lens for contextualizing the identified barriers and facilitators, aligning them with existing theoretical frameworks commonly used to study implementation in complex health systems.

**Table 2:** Sub−themes, categories, and codes

Factors influencing the delivery of family and community nursing care		
Sub-themes	Categories	Codes
**MACRO LEVEL**		
Wider environment	Policy and law	Regulatory legitimization of the FCN role (+)
	Community engagement	Community-based organization presence (+) Local political support and facilitation (+)
**MESO LEVEL**		
Organizational readiness to change	Mission alignment	Misalignment between strategic mandates and operational practices (−)
	Professional role legitimacy	Lack of recognition and understanding of the FCN educational and professional pathway (−)
	Leadership support	Active engagement of top managers in developing the FCN role (+)
Organizational support	Infrastructure and staffing	Lack of transportation resources (−) Lack of human resources (−) Inadequate nursing uniforms (−)
	Access to knowledge	Support for participation in specific training programs (+)
**MULTIPLE LEVEL**		
Financial resources	Financial incentives	Lack of financial incentives (−)
Feedback	Clinical documentation and evaluation	Heterogeneity and fragmentation of nursing documentation (−) Incomplete or insufficient performance evaluation systems (−)
	Information flows	Fragmentation of information flows (−)
Role recognition	Education and cultural models	Heterogeneity of FCN educational programs (−) Medically-centered model of care (−)
	Public and professional awareness	Lack of awareness of the FCN role among patients and caregivers (−) Limited awareness among healthcare professionals (−) Word-of-mouth supporting role recognition (+)
	Teamwork and professional interaction	Expansion of interprofessional collaboration opportunities (+) Collaboration dependent on individual professional engagement (−)

Legend: (+) = facilitator; (−) = barrier

#### Macro level

##### Wider environment

Six out of forty-one FCNs highlighted how external influences such as regulations and national policies drove healthcare organizations to adopt FCNs as a key profession to strengthen community-based care. The regulatory change occurred within a broader wave of initiatives prompted by the COVID-19 pandemic, which revealed the vulnerabilities of the primary care system and underscored the urgent need to reinforce community-based services.

Specifically, two participants described the importance of the National Recovery and Resilience Plan (NRRP), which outlines the structural, organizational, and technological standards to ensure homogeneous community-based care at the national level. The regulatory requirement to adopt national standards (Ministerial Decree 71/2021) served as a key enabler by providing a shared framework within which the FCN role is embedded. The subsequent Ministerial Decree 77/2022 further consolidated and legitimized the FCN role, formally recognizing it as a main element in delivering effective and integrated patient care at the community level.

From an operational perspective, this has facilitated the actual implementation of the role, prompting head nurses to support FCNs training and ensure their integration in community-based services in alignment with the regulatory standards. As one FCN stated: “*So*,* the fact that the role has been legally legitimized in some way motivates people and top management to activate it” (Int.01*,* F.*,* 50 aa)*. However, despite the regulatory framework, interviews with FCNs revealed a widespread awareness of the heterogeneity that still exists in the delivery of family and community nursing care across regions.

The Ministerial Decree 77/2022, while providing coherence and guidance for implementing the role, needed to be adapted to different local contexts, resulting in uneven application across regions. As one FCN shared: “*If you speak with 30 FCNs from 30 different healthcare organizations*,* you will get different versions of what they are doing*,* but the common foundation for all of them is that defined by Ministerial Decree 77/2022” (Int.01*,* F.*,* 50 aa)*.

Another facilitating factor was the active engagement of the community. In particular, five FCNs positively described their collaboration with local organizations, such as volunteer associations and local providers. These partnerships were perceived as essential for fostering community engagement, creating support networks that enhanced community care, improving access to services, and addressing complex socio-health needs more effectively. When collaborations were established based on shared aims and genuine participation between professionals and local actors, FCNs enabled more integrated and effective interventions to meet complex socio-health needs of the population, as highlighted by one participant: “*We work very well with the Intermunicipal Consortium for the Management of Social and Health Services; when we call*,* they are very helpful*,* and there is good collaboration” (Int.23*,* F.*,* 55 years old).*

At the same time, the support of local political institutions played an important role in promoting community-based care models. One FCN described the involvement of mayors as an enabling condition for achieving social recognition of the FCN role. In many cases, political support has facilitated the initiation of local initiatives, including home oxygen therapy programs involving clinical assessment of device appropriateness during home patient visits and community health events targeting individuals with chronic conditions. These initiatives strengthened the visibility of FCN roles and contributed to building trust between FCNs and the community.

As one FCN stated: “*It was really a great opening*,* because at the time the mayors would send an invitation letter to participate in this initiative*,* so that was very important*,* as was word-of-mouth” (Int. 18*,* F.*,* 31 aa)*.

#### Meso level

##### Organizational readiness to change

A common barrier reported by FCNs was the perceived misalignment between strategic leadership levels which defines the mandate to reorganize community-based care through regulatory directives and the managerial and operational levels responsible for executing these changes. This misalignment reflected limited organizational readiness to change, particularly where resource allocation was not aligned with strategic objectives. Participants highlighted that the lack of targeted investments such as staff training and the fragmented planning at the strategic level, hindered the effective implementation of the FCN role within community-based services. Moreover, participants also emphasized a structural weakness in healthcare organizations in translating strategic directives into practice. This misalignment has contributed to growing dissatisfaction among some healthcare professionals, who frequently reported being assigned tasks that diverged substantially from the core responsibilities of the FCN role. The frustration arising from this implementation gap between expectations and the actual organizational practices is reflected in the words of one FCN: “*In my view*,* the main challenge lies in the organization. On the one hand*,* nurse managers tell you: go ahead*,* push forward*,* there’s a mandate to implement the Family and Community Nurse*,* so feel free to act. However*,* on the other hand*,* every day they tell you: no*,* listen*,* today you have to do something else because there’s no other nurse available for that task” (Int.40*,* F.*,* 35 aa).*

Another barrier was the limited awareness of the FCN training pathway and the specific competencies that distinguish FCN roles from other nursing professionals. This knowledge gap directly influenced healthcare organizations’ ability to effectively integrate FCNs within existing care models.

When top-level and mid-level nurse managers including roles such as head nurses lacked full awareness of the FCN’s scope of practice and potential contribution, it became difficult to revise processes, roles, and support change at an operational level. One participant highlighted the need for better-informed managers to foster a deeper understanding of the FCN role: “*There are also organizational-level barriers*,* in the sense that I realize even the nurse manager or higher-level leaders seem to have a limited understanding of what the FCN can actually do. So*,* I would say there’s a real need to inform those in charge of organizational planning as well” (Int.37*,* F.*,* 38 aa).*

On the other hand, when nurse managers demonstrated a clear understanding of the FCN role, they were able to act as influence leaders in facilitating the integrations of the role into care models. Some participants reported that head nurses had proactively invested in building the operational capacity needed to support change. The words of one nurse reflected this active and intentional commitment, which resulted in tangible outcomes in terms of professional recognition and legitimization of the FCN role within services: “*The head nurse is someone who has received specific training on FCNs*,* and this is something I can clearly perceive…. I have always felt a lot of trust from them and strong support for the ideas and proposals I make” (Int.37*,* F.*,* 38 aa).*

##### Organizational support

Participants identified the shortage of instrumental and human resources as a significant barrier influencing the delivery of the FCN’s daily activities. In particular, the most reported hindering factor was the limited number of nurses engaged in community-based care. As one FCN stated: “*Lately*,* it has been difficult due to the current situation because resources are limited*,* staff are scarce*,* and we continue doing what we have always done” (Int.29*,* F.*,* 40 aa).* This shortage impacted not only the number of initiatives provided but also the quality of care, often leading to a prioritization of task-oriented interventions at the expense of the relational and educational aspects that characterize the FCN role.

Another barrier, reported by four nurses, concerned the limited availability of transportation provided to professionals working in community-based care. The lack of vehicles such as cars hindered the ability to ensure continuity of care, particularly in rural areas, which are more isolated from services, or geographically large districts. One FCN mentioned: *“Specifically*,* one barrier is the transportation*,* as we need to travel to various towns and there is strong competition for the available vehicles.” (Int.31*,* F.*,* 30 aa).*

Nurses also highlighted how this limitation impacted the organization of daily work, making it difficult to efficiently schedule and carry out home visits.

An additional limiting factor was the lack of appropriate uniforms for nurses working in community-based care. Two participants described how the uniforms provided were unsuitable for the climatic conditions and for the practical demands of fieldwork, leading FCNs to wear regular clothes instead. One FCN stated: *“We do not have a suitable uniform*,* we have the hospital uniform; for example*,* in the summer it was very hot; we wear a t-shirt that we had made ourselves*,* but it is not considered an official uniform…” (Int.36*,* F.*,* 26 aa).* This lack of appropriate uniform was perceived to influence the professional recognition of the FCN and made it more challenging to establish a relationship with patients. The absence of a standard uniform could lead to confusion regarding the FCN role and reduce the perceived sense of authority and trust that are essential in healthcare settings. As one FCN stated: “*We have to wear our own regular clothes this creates difficulties*,* especially when it comes to approaching patients.*” *(Int.31*,* F.*,* 30 aa).*

The only facilitating factor among these subthemes, was the organization’s active support for the continuous training of FCNs. The opportunity to access specialized training programs through company-funded scholarships, ongoing education, and peer learning initiatives was perceived to enhance nurses’ professional and interpersonal skills. Participants also emphasized that organizational commitment to supporting professional development significantly contributed to their overall job satisfaction and their perception of being valued in their role. As one FCN stated: “*I must say*,* in our healthcare organization*,* they (nurse managers) have always believed in our role and have invested a lot in training*,* so I can say that I am extremely satisfied.” (Int. 18*,* F.*,* 31 aa).*

#### Multiple Level

##### Financial resources

All participants identified the lack of adequate financial recognition commensurate with the competencies and responsibilities required by the FCN role as a major barrier. Appropriate financial remuneration was also perceived as a concrete expression of professional value and institutional recognition. Participants reported that, in many cases, FCNs were required to manage increasingly complex care situations, which entailed greater decision-making autonomy and broader clinical and ethical responsibilities. These demands were seen to have significant repercussions on personal and family lives, contributing to a perceived imbalance between role’s expectations and the corresponding financial remuneration. This sentiment was expressed by one FCN: *“While responsibilities continue to increase*,* remuneration does not rise proportionally. The role demands longer working hours and entails greater emotional and cognitive burden*,* ultimately reducing the time available for family life an imbalance for which financial remuneration offers insufficient compensation.” (Int. 39*,* M.*,* 41 aa).*

##### Feedback

All participants highlighted the heterogeneity and fragmentation of nursing documentation as a key barrier. Interview data revealed significant inconsistencies in the systems used to record the activities of FCN across different regions and within local healthcare organizations. The majority of nurses reported that the documentation tools currently in use had been originally developed for home care settings primarily aimed at recording task-oriented interventions and were therefore not fully aligned with the specific scope of FCN role, as illustrated by one FCN: “*We use an electronic health record to document the activities performed […]*,* but not everything related to the FCN role is captured within this system*,* as it was originally designed for home and palliative care.” (Int. 37*,* F.*,* 38 aa).*

To compensate for the lack of a documentation system capable of accurately and comprehensively capturing FCN activities, some participants reported supplementing official records with informal tools. One FCN referred to using Excel spreadsheets for this purpose: “*In the end*,* I can tell you that we do a lot. However*,* we still document everything in Excel*,* meaning we cannot report our work through official data streams or standardized indicators” (Int. 13*,* M.*,* 37 aa).*

This informal integration of documentation reflected a shared issue: the perceived invisibility of nursing work. This perceived invisibility was also linked, in participants’ accounts, to the way institutional performance was measured.

The current evaluation system was described as based on quantitative indicators such as the number of visits per patient and was perceived to inadequately capture the variety and complexity of services provided. For example, therapeutic education, counselling, or coordination with other healthcare professionals activities that are fundamental to the daily work of the FCNs were not adequately traceable within existing reporting systems. One FCN described: *“Another major difficulty is that nursing care provided to the family cannot be documented in any reporting system because our current systems only account for specific tasks. Therefore*,* everything I do*,* for example*,* in a nurse-led clinic*,* cannot be quantified” (Int. 01*,* F.*,* 50 aa).* Consequently, participants expressed concern that FCN contributions risked being marginalized within institutional monitoring systems, with potential concrete consequences in terms of how human resources were allocated. One FCN stated: “*The number of visits made is reported*,* which generates a coefficient that*,* based on the visit volume*,* can be higher or lower. If visits aren’t recorded*,* the coefficient stays low*,* which gives the impression that less work has been done. This also results in an inaccurate picture of how much staff is needed.” (Int. 39*,* M.*,* 41 aa).*

Finally, the fragmentation of nursing documentation was perceived to reflect a broader lack of integration in organizational information flows, which participants often described as not interoperable. The various systems did not communicate, forcing nurses to duplicate their documentation efforts. As a result, participants perceived that this situation reduced the time available for direct patient care and contributed to an increased sense of bureaucratic burden.

##### Role recognition

The interviews revealed significant relational and cultural barriers associated with the limited understanding and recognition of the FCN role among patients. This lack of awareness was perceived by participants as being closely linked to the primary care model, traditionally centered exclusively around the General Practitioner (GP), who continues to be perceived by the public as the only legitimate point of reference. As a result, some nurses reported encountering initial resistance from patients. The examples provided by participants pointed to a general confusion among citizens regarding the FCN role, which is sometimes misinterpreted as an administrative support function to the GP. One FCN clearly exemplifies this perception: *“Many citizens were confused and thought I was the GP’s secretary*,* so the role was not yet clearly understood” (Int.35*,* F.*,* 34 aa).*

This lack of clarity regarding the FCN role was also perceived to influence nurse-patient relationships. Participants reported that such misunderstandings could lead to attitudes of indifference or mistrust, limiting the development of an effective therapeutic alliance. One FCN described how some patients preferred to address the GP directly, refusing to engage with the nurse: *“The doctor has already explained everything to me*,* I’m fine*,* look*,* I will talk to the doctor about it” (Int.34*,* F.*,* 34 aa).*

However, once the initial phase of resistance was overcome, the role of FCN was highly appreciated. Three nurses described how informal and spontaneous mechanisms of service promotion such as word-of-mouth among citizens had contributed to a progressive increase of community awareness about the FCN initiatives: “*But let’s say that once we were able to organize meetings in the communities*,* they were very much appreciated by the population*,* and then they also spread the word” (Int.29*,* F.*,* 40 aa).*

Participants also reported that the lack of recognition of the FCN role was also evident in their interactions with colleagues without specific training in family and community nursing care. In some healthcare organizations, other nursing personnel did not fully understand the specific competencies of the FCN or the nature of the role itself. This lack of clarity led to ambiguity and uncertainty regarding the areas of intervention and the distinctive contribution that FCNs could offer within the healthcare team, as suggested by the words of one nurse: *“But it has happened that other colleagues ask me: ‘Does the family and community nurse do dressings?’ ‘What does the family and community nurse actually do?’ They don’t know what we do or what competencies we have acquired” (Int.37*,* F.*,* 38 aa).*

This barrier was reported to have significant operational consequences for the delivery of family and community nursing care. Participants noted that confusion regarding the FCN role reduced the potential for effective collaboration within the team, increasing the risk that the FCNs might be excluded from clinical decision-making or that their competencies would be undervalued.

The difficulty in recognizing the FCN role was further compounded by the lack of uniformity in the training pathways and role access criteria at the organizational level, which were reported to vary significantly across the regions. This heterogeneity was linked to the fact that, to practice as an FCN, professionals had completed specialized clinical courses or postgraduate degrees that differed in terms of educational commitment, delivery methods. These inconsistencies were perceived as contributing to disparities in both preparation and acquisition of the core competencies required to perform the role effectively. The lack of uniformity in training was perceived by participants as contributing not only to uncertainty among professionals, but also to fragmented perception of the FCN’s professional identity. As an FCN stated: “*For instance*,* depending on the region*,* access to FCN roles may require a master’s degree*,* a postgraduate degree or specialized clinical courses defined training. This inconsistency in educational requirements can lead to a fragmented perception of the FCN role and contribute to a lack of shared professional identity.*” (*Int.41*,* M.*,* 32 aa).*

Another dimension of the lack of recognition concerned relationships with other healthcare professionals, particularly GPs. Within a traditionally GP-centered primary care model, the nursing contribution to the patient care pathway struggled to gain visibility and integration, especially in the early phases of FCN implementation. The introduction of the FCN contributed to a shift in the organization of primary health care, leading to a redistribution of responsibilities that had previously been asymmetric and heavily favoring the GP, who was solely responsible for addressing the increasingly complex needs of patients. This change had, in some cases, generated resistance or mistrust, as described by an FCN who recalled the initial response of some GPs: *“When we started*,* it is undeniable that GPs had difficulty accepting our role because they saw us as somewhat intrusive in a field that had always been solely theirs.” (Int.16*,* F.*,* 48 aa).*

This difficulty, highlighted by the nurses, was reported to have practical implications, particularly for the development of shared care pathways with the GP. In some cases, interprofessional collaboration was hindered by the GP’s reticence to engage. Nurses describe how the quality and frequency of collaboration varied significantly depending on the individual GP’s openness. As a result, the human factor and the lack of formalized procedures represented a barrier, as noted by one nurse: “*We work with many GPs; sometimes I find a GP who collaborates*,* calls me*,* and even wants to do a joint visit*,* while other GPs may answer ‘yes*,* yes…’ when you call and then we don’t hear from them anymore.” (Int. 19*,* F.*,* 50 aa).*

Despite these challenges, participants also reported positive experiences of interprofessional collaboration, which were described as significant facilitating factors. Most nurses highlighted that increased opportunities for professional dialogue and collaboration with other specialists such as physiatrists and physiotherapists were crucial in ensuring more effective care, especially in complex cases requiring coordinated interventions. As one nurse described: *“We also have positive collaborations*,* for example with the physiatrist (for bedridden patients)” (Int.31*,* F.*,* 30 aa).*

## Discussion

This study explored barriers and facilitators perceived by FCNs in delivering community-based care to older adults in the Italian context. The main barriers identified were: a misalignment between strategic leadership and both managerial and operational levels; shortages of instrumental and human resources; heterogeneity and fragmentation of nursing documentation, which contributed to a lack of visibility of the FCNs’ contribution; inconsistencies in training pathways, leading to disparities in the development and consolidation of required competencies; and limited recognition of the FCN role among citizens, GPs, and nursing colleagues. Conversely, several key facilitators emerged: the existing regulatory framework and national policies particularly Ministerial Decree 77/2022 which formally recognized the FCN role; active support and collaboration from local organizations and political institutions; and the expansion of interprofessional collaborations aimed at strengthening care networks and improving the overall quality of patient care pathways.

Several recent Italian studies have examined the FCN in regional context [32, 33]. Notably, Clodig et al. [12] explored barriers and facilitators to the implementation of the FCN model, identifying similar organizational and systemic challenges, such as fragmented interprofessional collaboration, lack of shared documentation systems, limited leadership support, and low community awareness. While Clodig et al. [12] focused on the implementation phase, our study examined how these challenges continue to influence the actual delivery of FCN care in daily practice.

In our study, the most frequently reported topics that influencing the operational aspects of FCN were the integration of the role within healthcare organizations and the heterogeneity and fragmentation of nursing documentation. These two aspects directly influence the recognition of the FCN role and, consequently, impact the delivery of family and community nursing care.

About the first aspect, globally, and with relevance in Italy, healthcare organizations are experiencing a deep transformation in the delivery of community-based nursing care, viewing the introduction of new nursing roles as an opportunity to evolve toward care models that are more responsive to people’s needs and increasingly focused on proximity, continuity, and personalization of care. However, as emphasized by the WHO [34], such a shift requires strong organizational support, which is not always in place. Our findings reflect this tension: FCNs reported difficulties operating in contexts that struggle to sustain change, hindered by limited strategic managerial capacity and shortages of both instrumental and human resources. The WHO emphasizes the importance of obtaining organizational support for implementing community-based care models and adopting new nursing roles within primary care settings [34].

Consistent with the findings of Nilsen et al. [35], our results also underscore the challenges encountered when operating in organizational settings that are insufficiently prepared to support change. This organizational instability is further emphasized by Hosseinnejad et al. [36], who identified the lack of an adequately responsive structure as a key barrier. Such a limitation hampers the effective integration of community nursing services, ultimately delaying their implementation and consolidation within community-based care models [36].

In our study, participants reported that the lack of coherent resource planning contributes to organizational instability. Some interviews have explored the topic of organizational instability, FCNs are reported that they are assigned to tasks typically associated with integrated home care activities that diverge considerably from the advanced competencies and objectives defined for their role. This misalignment not only undermines the full potential of FCN practice but also contributes to feelings of demotivation. Similarly, Cheraghi et al. [37], emphasize that implementing a new role without sufficient organizational support can generate resistance to change, ultimately compromising the quality of care delivered.

This suggests that while a lack of organizational readiness can significantly undermine the integration of FCNs by generating disengagement, these very challenges may be mitigated or even transformed into opportunities through the presence of visionary, well-trained, and strategically engaged leadership, which emerges as a decisive enabling factor for navigating a fragmented healthcare system. In this regard, the interviews underscored that leadership support when exercised actively, strategically, and in alignment with the objectives of organizational change not only facilitates the integration process but also plays a pivotal role in fostering a culture of acceptance and innovation around new community-based roles. As noted by García-Sierra and Fernández-Castro [38], leadership support is a critical determinant for the effectiveness of innovative community-based care models.

The second aspect that emerged from the interviews is the absence of a structured nursing documentation system. Beyond its administrative purpose, documentation plays a crucial role in monitoring care activities and ensuring continuity of care by facilitating communication among healthcare professionals [39]. At the regional level, existing nursing documentation systems have been described as fragmented and insufficiently standardized, which limits the ability to accurately track nursing interventions and, as a result, diminishes the visibility of FCNs’ contributions [40]. This shortcoming has wider implications, as it complicates the demonstration of the role’s impact when activities performed in community settings are inadequately documented or lack transparency.

Effectively measuring this impact requires not only reliable and structured data but also the identification of nursing-sensitive outcomes that truly capture the value of FCN interventions. This challenge is especially pronounced in multiprofessional care environments, where outcomes typically result from collective efforts and are therefore not easily attributed to individual professionals [41, 42]. Recently, Russo et al. [42] have developed a study protocol aimed at designing a core set of nursing-sensitive outcomes specific to FCN practices, with the aim of more accurately assessing their contribution to health outcomes and enhancing their visibility in the healthcare system. Our findings highlighted how the lack of adequate documentation that allows the valorization of nursing interventions in community-based settings contributes to the widespread perception of limited recognition of the role at the national level. Similarly, Sánchez-Muñoz et al. [43] reported comparable perceptions in the Spanish context, where the limited recognition of the FCN role was closely linked to a lack of awareness regarding its scope, competencies, and potential impact. This limitation not only undermines the social visibility of the role but also weakens interprofessional collaboration. In some cases, this lack of recognition has led to the exclusion of nurses from clinical decision-making processes and the undervaluation of their professional competencies [43]. These lack of recognition issues appear to play a key role in shaping early interprofessional dynamics, particularly within primary care settings. In the early phases of FCN role implementation, collaboration between healthcare professionals encountered several difficulties, which hindered restricted FCN role integration into primary care teams. However, according to Gysin et al. [44] once the nature and added value of the role became clearer by GPs, a significant improvement in the patient’s care was observed. In according with our results, despite these initial challenges, interprofessional collaboration was identified as a key facilitating factor in the delivery of community-based nursing care [44]. Participants emphasized that working in close partnership with other healthcare professionals is crucial for strengthening care networks and improving care pathways particularly for older adults, who were identified as the primary target of FCN interventions. These findings are consistent with recent evidence from Scrimaglia et al. [11] and Torrens et al. [14], who underscore the central role of teamwork in the effective delivery of integrated nursing care.

### Implication for nursing management

The analysis of barriers and facilitators, based on the experiences of the professionals involved, suggests the need for targeted implementation strategies aimed at strengthening the nursing role in a community-based setting. To ensure effective and high-quality family and community nursing care, healthcare systems must invest in developing nursing leadership capable of supporting the role integration process and promoting interprofessional networks. Leadership that values the specific contribution of the nursing profession and fosters a supportive work environment is crucial to overcoming persistent obstacles, such as instrumental and human shortages and organizational resistance to change. This would help enhance continuity of care and the effectiveness of services, particularly in managing vulnerable populations with complex and chronic needs.

### Limitations

This study has several limitations. First, the purposive and snowball sampling methods may have led to the selection of particularly motivated nurses, potentially excluding those who might hold a different view on the role of FCNs. While the inclusion of professionals from four Italian regions (representing the North, Center, and South) was intended to ensure national representativeness, this approach may limit the transferability of findings. Another limitation pertains to the data collection method: the interviews were conducted via VoIP tools, which may have reduced the depth of the interaction between the interviewer and interviewee compared to face-to-face meetings. Additionally, while the verbatim translation from Italian to English was performed accurately, some linguistic or cultural nuances inherent to the Italian language may have been lost.

## Conclusion

The results of this qualitative descriptive study highlighted which factors at the macro, meso, and multiple levels, influence the delivery of family and community nursing care. The perceived facilitators are mainly associated with the availability of supportive national policies, community-based organizations, and local political institutions. Barriers are more prominent and predominantly relate to fragmented documentation, inadequate evaluation processes, and limited role recognition.

Although national policies have contributed to legitimizing the FCN role, its implementation is still hindered by a lack of alignment between strategic leadership levels and the available instrumental and human resources. The study emphasizes the need for targeted interventions to strengthen the implementation of the FCN role, ensuring greater coordination between policy directives, workforce planning and organizational directives. Moreover, it is crucial to promote the development of a professional and organizational culture that adequately recognizes and values the contribution of FCNs within the primary care system, fostering a participatory approach that actively engages citizens. To support the consolidation of the nursing profession and the advancement of its competencies, future research should focus on monitoring the progress of the role’s implementation at the national level. Given the challenges related to documenting nursing activities, future researchers could aim to develop data sets consistent with the professional role, thereby enabling the visibility of activities that remain largely unseen.

## Data Availability

The datasets generated during and/or analyzed during the current study are available from the corresponding author on reasonable request.

## Abbreviations

FCN: Family and Community Nurse
WHO: World Health Organization
AGENAS: National Agency for Regional Health Services
SRQR: Standards for Reporting Qualitative Research
SD: Standard Deviation
VoIP: Voice-over Internet Protocol
NRRP: National Recovery and Resilience Plan
GP: General Practitioner
